# NLRP3 Inflammasome and Mineralocorticoid Receptors Are Associated with Vascular Dysfunction in Type 2 Diabetes Mellitus

**DOI:** 10.3390/cells8121595

**Published:** 2019-12-08

**Authors:** Nathanne Santos Ferreira, Thiago Bruder-Nascimento, Camila André Pereira, Camila Zillioto Zanotto, Douglas Silva Prado, Josiane Fernandes Silva, Diane Meyre Rassi, Maria Cristina Foss-Freitas, Jose Carlos Alves-Filho, Daniela Carlos, Rita de Cássia Tostes

**Affiliations:** 1 Department of Pharmacology, Ribeirao Preto Medical School, University of Sao Paulo, Ribeirao Preto 14040-900, Brazil; bruderthiago@yahoo.com.br (T.B.-N.); mila_cap@yahoo.com.br (C.A.P.); camizanotto@gmail.com (C.Z.Z.); pradodds@gmail.com (D.S.P.); jozibio@gmail.com (J.F.S.); dianerassi@usp.br (D.M.R.); jcafilho@fmrp.usp.br (J.C.A.-F.); 2 Department of Clinical Medicine, Ribeirao Preto Medical School, University of Sao Paulo, Ribeirao Preto 14040-900, Brazil; crisfoss@fmrp.usp.br; 3 Department of Biochemistry and Immunology Ribeirao Preto Medical School, University of Sao Paulo, Ribeirao Preto 14040-900, Brazil; danicar@usp.br

**Keywords:** type 2 diabetes, aldosterone, mineralocorticoid receptor, NLRP3 inflammasome

## Abstract

Aldosterone excess aggravates endothelial dysfunction in diabetes and hypertension by promoting the increased generation of reactive oxygen species, inflammation, and insulin resistance. Aldosterone activates the molecular platform inflammasome in immune system cells and contributes to vascular dysfunction induced by the mineralocorticoid hormone. It is unclear as to whether the NLRP3 inflammasome associated with the mineralocorticoid receptor contributes to vascular dysfunction in diabetic conditions. Here, we tested the hypothesis that an excess of aldosterone induces vascular dysfunction in type 2 diabetes, via the activation of mineralocorticoid receptors (MR) and assembly of the NLRP3 inflammasome. Mesenteric resistance arteries from control (db/m) and diabetic (db/db) mice treated with vehicle, spironolactone (MR antagonist) or an NLRP3 selective inhibitor (MCC950) were used to determine whether NLRP3 contributes to diabetes-associated vascular dysfunction. Db/db mice exhibited increased vascular expression/activation of caspase-1 and IL-1β, increased plasma IL-1β levels, active caspase-1 in peritoneal macrophages, and reduced acetylcholine (ACh) vasodilation, compared to db/m mice. Treatment of db/db mice with spironolactone and MCC950 decreased plasma IL-1β and partly restored ACh vasodilation. Spironolactone also reduced active caspase-1-positive macrophages in db/db mice, events that contribute to diabetes-associated vascular changes. These data clearly indicate that MR and NLRP3 activation contribute to diabetes-associated vascular dysfunction and pro-inflammatory phenotype.

## 1. Introduction

Diabetes mellitus (DM) is a chronic disease that affects 425 million people, and it is expected that by 2035, 592 million people will live with the disease [1]. Vascular complications associated with DM increase the risk of cardiovascular events and end-stage renal disease. Additionally, patients with diabetes are twice as likely to develop hypertension compared to the non-diabetic population [2]. Diabetes and hypertension are two of the more common coexisting diseases worldwide and contribute to the acceleration of microvascular and macrovascular complications. Hyperglycemia, insulin resistance, reactive oxygen species (ROS) and inflammation are key mechanisms involved in the pathogenesis and vascular complications associated with DM and hypertension [3,4,5,6].

Aldosterone, a steroid hormone, regulates the osmotic balance between water and electrolytes in the body [7,8,9]. However, an excess in aldosterone leads to vascular dysfunction, increased ROS generation and inflammation, contributing to the development of cardiovascular diseases in diabetic patients and in experimental animal models of diabetes [10,11,12,13,14,15]. An excess of aldosterone is a cardiovascular risk not only in diabetes, but also in hypertension, stroke, coronary artery disease and congestive heart failure [16,17]. Accordingly, the use of mineralocorticoid receptor (MR) antagonists increases the life expectancy of patients with severe heart failure, ameliorates nephropathy in type 2 diabetes, and reverses hypertension- and diabetes-associated vascular dysfunction [13,14,15,18].

Inflammation plays a critical role in initiating the vascular alterations associated with aldosterone and MR activation [19,20,21]. Many studies in the last decade have consistently shown that inflammation, alongside the function of the immune system can lead to target-organ injury in cardiovascular and metabolic diseases. Aldosterone stimulates the adaptive [20,22,23] and innate immune systems, increasing the activity of pro-inflammatory transcription factors, the production of adhesion molecules, chemokines, interleukins (IL) and pro-inflammatory cytokines [24,25,26].

We demonstrated that the NLRP3 (NOD-, LRR- and pyrin domain-containing protein 3) platform—a member of the Nod-like receptors (NLRs), that activates caspase-1 and the proteolytic cleavage of pro-IL-1β and pro-IL-18 to their active forms of IL-1β and IL-18, respectively [26]—contributes to vascular injuries induced by aldosterone [27]. Different groups have also provided evidence that aldosterone excess contributes to increased hypertension and fibrosis, and induces renal tubular cell injury via MR and mitochondrial ROS-mediated NLRP3 activation [18,28,29]. However, it is unknown whether aldosterone induces vascular dysfunction and complications in diabetes via NLRP3 inflammasome activation. 

We tested the hypothesis that vascular dysfunction in type 2 diabetes is mediated by the activation of MR and assembly of the NLRP3 inflammasome. The aims of this study were to investigate whether vascular dysfunction and inflammatory phenotypes in diabetes are associated with increased levels of aldosterone. More specifically, we determined the involvement of mineralocorticoid and NLRP3 receptors in vascular dysfunction in mice with type 2 diabetes. 

## 2. Materials and Methods

### 2.1. Animals

All experimental protocols were performed in accordance with the ARRIVE Guidelines (Animal Research: Reporting of in Vivo Experiments) and National Institutes of Health (NIH) Guide for the Care and Use of Laboratory Animals and were approved by the local Committee for Animal Research (Protocol No. 80/2015—Ribeirao Preto Medical School—University of Sao Paulo, Ribeirao Preto, Brazil). 

Male db/db mice and their respective controls (db/m mice)—purchased from The Jackson Laboratory, Bar Harbor, Maine, USA—were used at 10–14 weeks old. Male C57BL/6 wild-type (WT) mice at 8–10 weeks old were also utilized. The mice were allocated a room with a controlled temperature (22 ± 2 °C) and controlled humidity (50% ± 10%). Ventilated cages (4 mice/cage—600 cm^2^) were used, and day/night [12/12 h] cycles were respected. Food (Nuvilab^®^ mice chow pellets, Nuvital, Curitiba, Brazil) and potable drinking water were provided ad libitum.

The control and db/db mice received either the MR antagonist spironolactone treatment (50 mg/kg body weight/day, mixed with food, for six weeks) or vehicle. Body weight, food intake and glucose levels were monitored. The glycaemia was evaluated at weeks 1, 3 and 6 during the treatment (Accu-Chek Active^®^ Roche Diagnostics, Mannheim, Germany). 

Non-diabetic and diabetic mice were also treated with the NLRP3 selective inhibitor MCC950 (10 mg/kg/day via intraperitoneal injections) or a vehicle for 2 weeks. Glycaemia levels were monitored every 2 days. After two weeks, euthanasia was performed under anesthesia (2% isoflurane vaporized with oxygen) by cervical dislocation, and blood and tissues were collected.

### 2.2. Vascular Reactivity

For vascular reactivity assay, resistance mesenteric arteries without fat and connective tissue were used. Krebs–Henseleit-modified solution was used [(in mM): 130 NaCl, 4.7 KCl, 14.9 NaHCO_3_, 1.18 KH_2_PO_4_, 1.17 MgSO_4_ 7H_2_O, 5.5 glucose, 1.56 CaCl_2_ 2H_2_O, and 0.026 EDTA]. The pH was maintained using 5% CO_2_ and 95% O_2_. The rings of second-order arteries were coupled to an isometric myograph (Mulvany–Halpern model 610M; Danish Myo Technology, Copenhagen, Denmark) and data acquired by PowerLab 8/SP (ADInstruments, Sao Paulo, Brazil). The use of potassium chloride (KCl-120 mmol/L) indicated the arterial integrity, and the endothelium integrity was tested using an endothelium-dependent agonist (acetylcholine—ACh, 10 μM) on pre-contracted vessels (phenylephrine—PE, 3 μM). Concentration–response curves to PE and ACh (10^−10^ to 3 × 10^−5^ M) were performed in the mesenteric arteries of mice from each of the various experimental groups. 

### 2.3. IL-1β and Aldosterone Measurement

The IL-1β production in the mice of each experimental group was determined using ELISA (BD Biosciences. San Jose, CA, USA). The serum aldosterone of the mice of each experimental group was measured using aldosterone ELISA kits (Abcam, Cambridge, UK).

### 2.4. Western Blotting

The expression of caspase-1 and IL-1β in the mesenteric arteries was determined using protein analysis. Tissues from mice were frozen and the proteins collected. The proteins were separated using 12% polyacrylamide gels. Nitrocellulose membranes were used and blocked for 1 h at room temperature with bovine serum albumin (BSA) in Tris-buffered saline solution with Tween (0.1%). The antibodies (at the indicated dilutions) against NLRP3 (1:500, R&D Systems, MAB7578, Minneapolis, MI, USA): caspase-1 (1:1000, Imgenex, NB100-56565F, Centennial, UK); IL-1β (1:500, Santa Cruz Biotechnology, sc-7884, Bath, UK); GAPDH (1:10000 Sigma, G9545-100UL, Saint Louis, MO, USA); and β-actin (1:3000, Cell Signaling, 4967L, Woburn, UK) were incubated overnight at 4 °C. The secondary antibodies were incubated for 1 h: anti-Rat (1-1000, Sigma-Aldrich, A9037, Saint Louis, MO, USA); anti-Rabbit (1-3000, Abcam, ab6703, Cambridge, UK); and anti-Mouse (1-3000, Abcam, ab6789, Cambridge, UK) signals were revealed through chemiluminescence using Luminata™ Forte Western HRP Substrate (Millipore^®^, Burlington, Massachusetts, MA, USA), and the images were captured in a ImageQuant 350 Photodocumentation system (GE Healthcare^®^, Piscata Way, NJ, USA). The Image J^®^ program (version 1.51J8, Wayne Rasband, National Institutes of Health, Bethesda, MD, USA) was used to quantify the images, and the results were expressed as arbitrary units (A.U.).

### 2.5. Caspase-1 Activity by Flow Cytometry Analysis

Cells from the peritoneal lavage of control and db/db mice were used. A FAM-FLICA caspase-1 assay (FAM-YVAD-FMK) was used to stain the cells for 1 h (Immunochemistry Technologies, Bloomington, MN, USA). FACS Calibur (BD Biosciences, San Jose, CA, USA) was utilized to acquire samples, and FlowJo software (FlowJo Engine v4.000770 FlowJo 10.5.3, Tree Star, Ashland, OR, USA) was used to process and analyze the data. During peritoneal lavage, all cells were stained, but only the F4/80 positive macrophages were used in the analysis (Figure S1).

### 2.6. Lucigenin

Superoxide anion generation was evaluated in the mesenteric beds of the mice using a chemiluminescence assay. Lucigenin and NAD(P)H were used as the electron acceptor and substrate, respectively. Mesenteric arteries from control and db/db mice were incubated with vehicle or MCC950 (10^−6^ M) for 1 h. The mesentery arteries from C57BL/6 were stimulated with aldosterone (10^−7^ M – 30 min) and an MR antagonist or NLRP3 inhibitor (10^−6^ M) for 1 h. After stimulus, the arteries were transferred into glass tubes containing 950 μL HANK’S solution [(in mM): NaCl 120, CaCl_2_ 1,6, KCl 5, MgCl_2_ 6 H_2_O 1, NaH_2_PO_4_ 0,5, glucose 10, HEPES 10] and 5 μL of lucigenin (5 μM) for basal luminescence reading. After the baseline reading, 50 μL of NAD(*p*)H (100 μM) was added to the tube, and superoxide anion generation was quantified using the Line TL Tube Luminometer (Titertek-Berthold^®^, Pforzheim, Germany). Superoxide anion generation was expressed in RLU (relative units of luminescence)/dry weight (g).

### 2.7. Drugs

Phenylephrine, acetylcholine, aldosterone, spironolactone and MCC950 (CP-456773 sodium salt) were purchased from Sigma Chemical Co (St. Louis, MO, USA).

### 2.8. Data Analysis and Statistical Procedures

The PE-induced contractions are represented as a percentage of 120 mM KCl-induced contractions. The KCl response was similar between the groups (*p* > 0.05). The ACh-induced vasodilation is expressed as a percentage of vasoconstriction to PE. The sigmoid curves were fitted using the Prism software, version 6.0 (GraphPad Software Inc., San. Diego, CA, USA), which was also used for the non-linear regression analysis and the determination of *p*D_2_ (defined as the negative logarithm of the EC_50_ values) (Table S1) and maximal response (Rmax) values (Table S2). Statistical analyses were performed by one-way or two-way ANOVA, followed by Bonferroni multiple comparisons post-test, and the *p* values accepted were similar or less than 0.05. These data are presented as mean ± SEM, with N representing the number of animals used. 

## 3. Results

### 3.1. Spironolactone Treatment Reduces Vascular Dysfunction and Inflammasome Activation in db/db Mice

**Figure 1:**
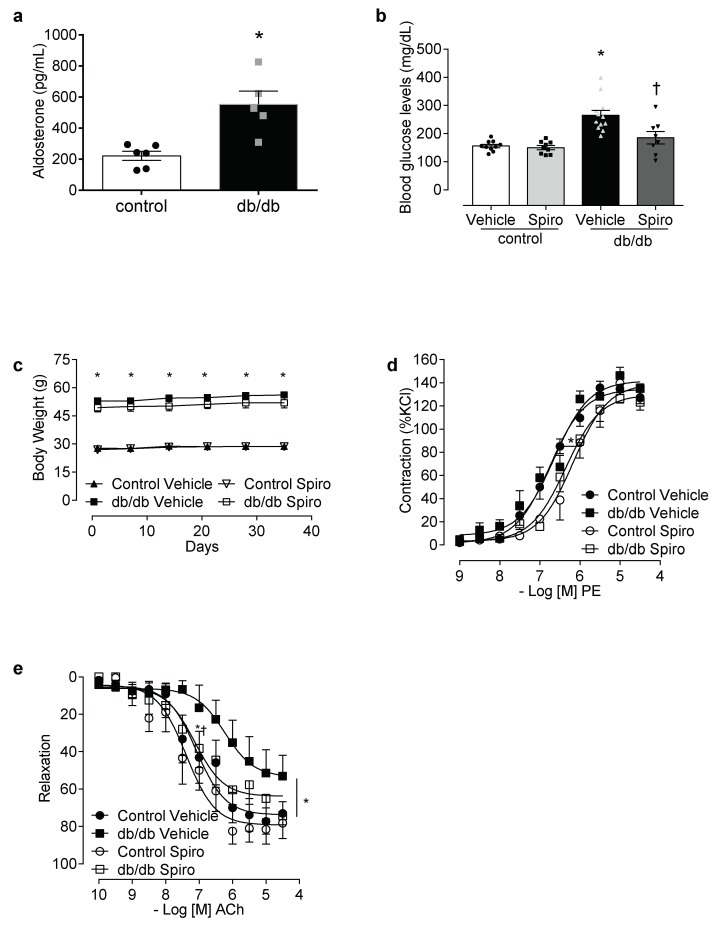
Mineralocorticoid receptors (MR) activation contributes to increased blood glucose levels and vascular dysfunction in diabetes. Aldosterone levels in control and db/db mice (**a**), plasma glucose levels at 6 weeks of treatment (**b**), body weight (**c**), contractile responses to phenylephrine (**d**) and relaxation to acetylcholine (**e**) of mesenteric arteries in control and db/db mice treated with a vehicle or spironolactone for 6 weeks. Data represent the mean ± S.E.M (n = 4–12 mice per group). In scatterplot with bar graphs, each symbol corresponds to one animal (a—circle: control vehicle; square: db/db vehicle; b—circle: control vehicle; square: control spironolactone; triangle: db/db vehicle and inverted triangle: db/db spironolactone). Student t-test and two-way ANOVA with Bonferroni post-test, *p* < 0.05 * db/db vehicle vs. control (**a**–**e**); † db/db spironolactone vs. db/db vehicle (**b**,**e**). Spiro: Spironolactone, PE: phenylephrine, ACh: acetylcholine.

**Figure 2:**
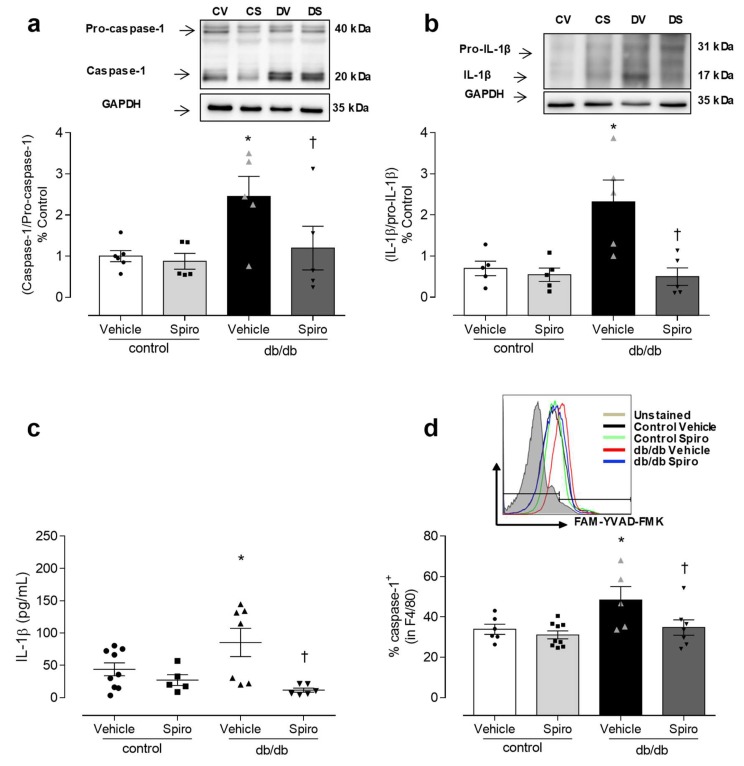
MR activation contributes to inflammasome activation in diabetes. Representative immunoblotting and corresponding graphs depicting vascular expression of caspase-1 (**a**) and IL-1β (**b**), determined by Western blot, in mesenteric arteries of control and db/db mice treated with spironolactone or a vehicle for 6 weeks. Plasma levels of the cytokine IL-1β (**c**), and percentage of caspase-1 activity in macrophages of peritoneal lavage (**d**), from vehicle- and spironolactone-treated control and db/db mice. These data represent the mean ± S.E.M (n = 5-8 mice per group). In scatterplot with bar graphs, each symbol corresponds to one animal (**a**–**d**—circle: control vehicle; square: control spironolactone; triangle db/db vehicle and inverted triangle: db/db spironolactone). Two-way ANOVA with Bonferroni post-test, *p* < 0.05 * db/db vehicle vs. control (**a**–**d**); † db/db spironolactone vs. db/db vehicle (**a**–**d**). Spiro: Spironolactone, CV: control vehicle, CS: Control Spironolactone, DV: db/db vehicle, DS: db/db Spironolactone.

Aldosterone excess in diabetes is linked to the activation of MR and inflammatory processes [13,14,30,31,32,33]. To determine the contribution of aldosterone and MR toward inflammasome activation, db/db mice were treated with spironolactone. The db/db mice displayed increased aldosterone levels (Figure 1a), increased blood glucose levels, and increased body weight compared to the control mice. Treatment with spironolactone for 6 weeks reduced blood glucose levels in the db/db mice (Figure 1b), but did not alter body weight in either the control or the db/db mice (Figure 1c). The PE-induced vasocontractions were similar between both the vehicle-treated control and the db/db mice. However, spironolactone treatment decreased the phenylephrine potency in arteries from both the control and the db/db mice (Figure 1d, Tables S1 and S2). Mesenteric resistance arteries taken from the db/db mice exhibited reduced ACh-induced dilation, which was abolished by spironolactone treatment (Figure 1e, Tables S1 and S2). The expression of active caspase-1 and mature IL-1β was increased in the db/db mesenteric arteries. Spironolactone treatment reduced the activation of caspase-1 (Figure 2a) and mature IL-1β content (Figure 2b) in arteries taken from mice with type 2 diabetes. The db/db mice exhibited increased plasma IL-1β levels, which were decreased following treatment with the MR receptor antagonist (Figure 2c).

Considering the importance of macrophages in inflammatory responses, the potential of aldosterone to activate the macrophages inflammasome in db/db mice, and the effect of MR antagonist treatment on inflammasome activation in the macrophages of db/db mice were both determined. In the peritoneal lavage, the number of active caspase-1-positive macrophages was increased in the db/db mice compared to in the control mice. Spironolactone treatment reduced the number of active caspase-1-positive-macrophages in the db/db mice (Figure 2d), supporting the theory that aldosterone activates the NLRP3 inflammasome in the immune cells of mice with type 2 diabetes.

These results suggest that both aldosterone and MR activation contributes to vascular damage, and to the activation of the NLRP3 inflammasome in the vasculature and macrophages of db/db mice.

### 3.2. NLRP3 Inhibition Attenuates Vascular Dysfunction and Decreases Reactive Oxygen Species Generation in Mesenteric Arteries of db/db Mice

**Figure 3:**
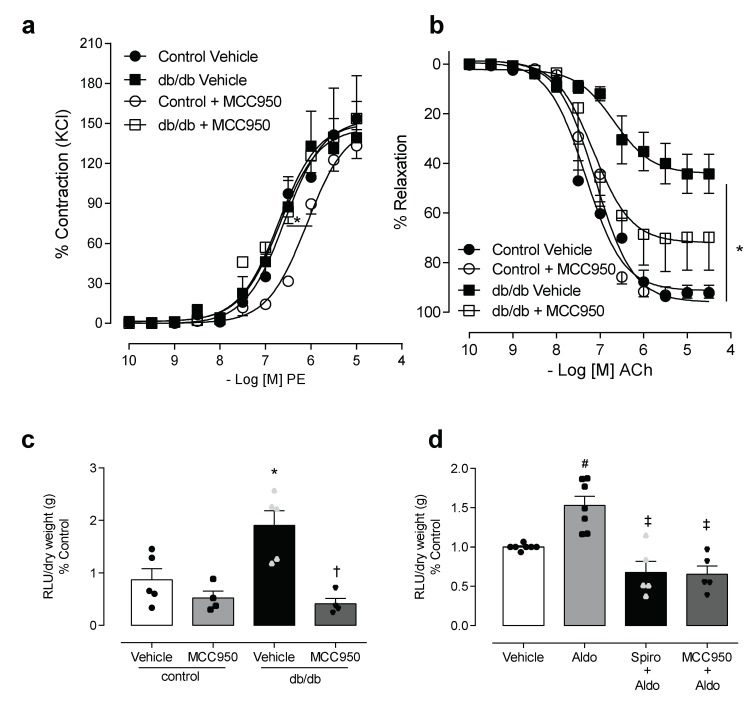
NLRP3 activation contributes to aldosterone-induced oxidative stress. The effects of MCC950 on vascular reactivity and reactive oxygen species (ROS) generation were assessed in mesenteric arteries from control and db/db mice. Contractile responses to phenylephrine (**a**), relaxation responses to acetylcholine (**b**) and ROS generation (**c**) in mesenteric arteries, from control and db/db mice after 1 h of incubation with vehicle or the NLRP3 inhibitor. ROS generation was also determined in mesenteric arteries from control C57BL/6 mice, stimulated with aldosterone (10 µM for 30 min) in the presence of spironolactone (1 µM for 1 h) or MCC950 (1 µM for 1 h) (**d**). These data represent the mean ± S.E.M (n = 4–7 mice per group). In scatterplot with bar graphs, each symbol corresponds to one animal (**c**–circle: control vehicle; square: control spironolactone; triangle: db/db vehicle and inverted triangle: db/db spironolactone; **d**—circle: control vehicle; square: aldosterone; triangle: spironolactone+aldosterone and inverted triangle: MCC950+aldostorone). Two-way/One-way ANOVA with Bonferroni post-test, *p* < 0.05 * control MCC950 vs. control vehicle (**a**); * db/db vehicle vs. control vehicle (**b**–**d**); † db/db spironolactone vs. db/db vehicle and # aldosterone vs. vehicle, ‡ spironolactone+aldosterone and MCC950+aldostorone vs. aldosterone. PE: phenylephrine, ACh: acetylcholine: MCC950: NLRP3 inhibitor, RLU: relative light units.

To determine the involvement of NLRP3 in diabetes-associated vascular dysfunction, we used an NLRP3 inhibitor, MCC950. The vascular function of the control and diabetic groups was evaluated in resistance mesenteric arteries, incubated for 1 h with MCC950. No changes in maximal contractile response to PE were observed between the control and diabetic groups in the presence of vehicle or MCC950. However, the presence of MCC950 shifted the potency of the PE response to the right in the control group (Figure 3a, Tables S1 and S2). Endothelium-dependent relaxation induced by ACh, as previously described, was reduced in db/db mice when compared to the control. The use of the NLRP3 inhibitor in vitro partially attenuated the impairment of the vasodilation response in the mice with diabetes (Figure 3b, Tables S1 and S2). Since (i) aldosterone has pro-oxidative and pro-inflammatory effects, (ii) aldosterone-induced ROS generation is linked to vascular dysfunction in diabetes [13,14,18,34,35]; and (iii) ROS are one of the main activators of NLRP3 inflammasome [18,36], we tested the effect of the selective inhibitor of the NLRP3 receptor on ROS generation in mesenteric arteries taken from mice with diabetes, as well as in arteries taken from control mice that were stimulated with aldosterone. The effects of MCC950 on ROS generation in mesenteric arteries of both control and db/db mice were assessed using lucigenin assay. The NLRP3 inhibitor abolished the increased vascular ROS generation in db/db mice (Figure 3c). MCC950 also attenuated aldosterone-induced ROS generation in the mesenteric arteries of the control mice (Figure 3d).

### 3.3. In Vivo Treatment with NLRP3 Inhibitor Reduces Vascular Dysfunction and Decreases IL-1β Levels in db/db Mice

**Figure 4:**
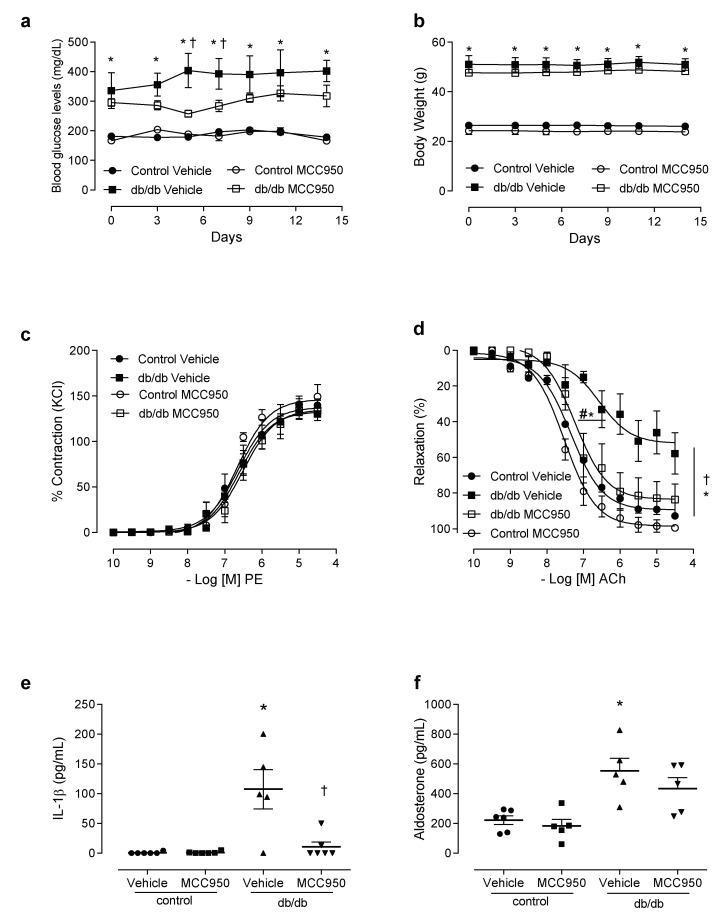
NLRP3 receptor contributes to vascular dysfunction in diabetes. Plasma glucose levels (**a**), body weight (**b**), contractile responses of mesenteric arteries to phenylephrine (**c**) and relaxation responses to acetylcholine (**d**), plasma levels of IL-1β (**e**) and aldosterone (**f**) in control and db/db mice treated with vehicle or MCC950 for 14 days. These data represent the mean ± S.E.M (n = 4–7 mice per group). Two-way/One-way ANOVA with Bonferroni post-test, *p* < 0.05 * db/db vehicle vs. control vehicle; † db/db MCC950 vs. db/db vehicle. MCC950: NLRP3 inhibitor, PE: phenylephrine, ACh: acetylcholine.

To determine the direct involvement of NLRP3 in diabetes-associated vascular dysfunction, db/db mice were treated for 2 weeks with a specific NLRP3 inhibitor. Treatment with MCC950 did not reduce blood glucose levels (Figure 4a), glucose tolerance (Figure S2) or body weight (Figure 4b) in the control or the db/db mice. MCC950 did not alter PE-induced contractions in mesenteric arteries of the experimental groups (Figure 4c, Tables S1 and S2), either. However, decreased ACh-induced dilation in mesenteric resistance arteries from db/db mice was prevented by treatment with MCC950 (Figure 4d). Treatment with the NLRP3 inhibitor also reduced the plasma levels of IL-1β in the db/db mice (Figure 4e). MCC950 did not reduce aldosterone levels (Figure 4f).

## 4. Discussion

This study demonstrates the important role of NLRP3 inflammasome activation and its association with aldosterone in diabetes vascular dysfunction. In particular, it shows that (i) db/db mice display increased levels of aldosterone as well as increased caspase-1 activation and IL-1β production in mesenteric arteries; (ii) spironolactone, a MR antagonist, prevents these events; (iii) NLRP3 inhibition in vitro and in vivo reverses endothelial dysfunction in db/db mice.

Plasma aldosterone levels—augmented in diabetic and hypertension patients and experimental models of diabetes—contribute to the increased expression of inflammatory markers, such as TNF-α, chemotactic protein macrophage, transforming growth factor-beta and IL-1β [18,30,32,33,37]. Additionally, aldosterone stimulates proinflammatory transcription factors, such as nuclear factor kappa B (NF-kB) (30-33). NF-kB induces the production of adhesion molecules, chemokines and cytokines (34), including pro-IL-1β and pro-IL-18, which are converted to their mature forms after the activation of the inflammasome. Aldosterone, itself, activates the inflammasome in immune cells by activating NF-kB and producing ROS, as we recently showed [18] in an experimental model of aldosterone-inducing hypertension.

The involvement of MR in inflammatory processes is associated with the activation of MR in cells of the immune system. Accordingly, animals with a deletion of MR in their macrophages are protected from cardiac fibrosis and increased blood pressure, induced by treatment with deoxycorticosterone and salt (DOCA-salt) for 8 weeks [30,31]. In addition, MR deficiency in myeloid cells mimics the effects of MR antagonists and protects against cardiac hypertrophy, fibrosis, and vascular damage caused by N omega-Nitro-L-arginine methyl ester hydrochloride (L-NAME) and angiotensin II [38]. Here, we demonstrated that db/db mice display increased caspase-1 activity in macrophages, and that spironolactone treatment reduces this activity. Additionally, spironolactone also reduced inflammatory cytokines, improved endothelium-dependent vasodilation, and reduced blood glucose levels in the type 2 diabetes model mice. 

Doi et al. (2014) reported that the immunosuppressive drug mizoribine reduces blood pressure levels and renal inflammasome markers such IL-1β, caspase-1 and NLRP3 in DOCA/salt hypertensive rats [39,40]. Mice with DOCA/salt-induced hypertension exhibited increased renal expression of the inflammasome components, and treatment with an inflammasome inhibitor reduced hypertension and inflammatory markers. Similarly, ASC-/- mice submitted to DOCA/salt hypertension also displayed blunted pressor responses, and did not exhibit increased expression of renal inflammatory markers, suggesting an important role of inflammasome components in the development of hypertension and hypertension-associated renal abnormalities.

In the vasculature, mature IL-1β, the final product of NLRP3 inflammasome activation, directly influences vascular responses. The incubation of aortic rings from stroke-prone, spontaneously hypertensive rats with IL-1β for 1 h significantly increases contractility via the activation of cyclooxygenase- and Src-kinase-related signaling pathways [40]. In addition, vascular dysfunction in an experimental model of type 1 diabetes relies on IL-1R receptor activation, since treatment with anankira (an IL-1 receptor antagonist) reduces endothelial dysfunction, vascular oxidative stress and inflammation [41], suggesting that the activation of the inflammasome contributes to further vascular changes. The activation of the components of the innate immune system in the vasculature and immune cells of animals with type 2 diabetes, as shown in the present study, confirms the key role of the immune system in relation to vascular injury in cardiovascular/metabolic diseases.

Inflammasome activation in the macrophages of db/db mice impairs wound healing, and reduces the expression of endogenous inflammasome inhibitors, such as PI-9 and caspase-12. Additionally, the transplantation of bone-marrow-derived cells, from NLRP3 or caspase-1 knockout mice to db/db mice, improves both wound healing and the participation of macrophages in the healing process [42]. These data show that the inhibition of NLRP3 prevents vascular dysfunction and the expression of inflammatory markers in the vasculature of db/db mice, developing our understanding of the implications of NLRP3 activation in diabetes.

The important effect associated with vascular dysfunction is ROS generation. Our group showed that spironolactone reduced ROS generation, improved vascular dysfunction and increased antioxidant enzymes in this diabetes model [13]. The decreased superoxide anion generation in arteries incubated with NLRP3 receptor observed in the present study could be associated with the improved vasodilation observed in the arteries of db/db mice incubated or treated with MCC950. In NLRP3 knockout mice with aldosterone-induced hypertension, vascular dysfunction, oxidative stress and caspase-1 activity were all reduced [18], further supporting the hypothesis that decreased NLRP3 receptor activation reduces IL-1β production, ROS generation and NLRP3 activation-associated inflammatory processes.

## 5. Conclusions

In conclusion, our study shows an association between vascular dysfunction in type 2 diabetes, and NLRP3 inflammasome and MR receptor activation. NLRP3 activation and associated inflammatory events may be especially important in conditions associated with increased aldosterone levels, such as obesity, metabolic syndrome, and arterial hypertension.

## Supplementary Materials

The supplementary materials are available online at https://www.mdpi.com/2073-4409/8/12/1595/s1.
