# Supplementary material for: NLRP3 Inflammasome and Mineralocorticoid Receptors Are Associated with Vascular Dysfunction in Type 2 Diabetes Mellitus
Source: Cells. 2019 Dec 8;8(12):1595. doi: 10.3390/cells8121595 (PMC6952964; doi:10.3390/cells8121595)
Supplement: Supplementary file 1 [file cells-08-01595-s001.pdf]

NLRP3 inflammasome and mineralocorticoid receptor are associated with vascular dysfunction in type 2 diabetes mellitus

Nathanne S Ferreira[1*], Thiago Bruder-Nascimento[1], Camila A Pereira[1], Camila Z Zanotto[1], Douglas S Prado[1], Josiane F Silva[1], Diane M Rassi[1], Maria Cristina Foss-Freitas[2], Jose C Alves-Filho[1,3], Daniela Carlos[3], Rita C Tostes[1*]

Departments of [1]Pharmacology, [2]Clinical Medicine, Division of Endocrinology, and [3]Biochemistry and Immunology, Ribeirao Preto Medical School, University of Sao Paulo, Ribeirao Preto, SP, Brazil.

***Corresponding authors**
Rita C Tostes / Nathanne dos Santos Ferreira
Department of Pharmacology
Ribeirao Preto Medical School, University of Sao Paulo,
Av Bandeirantes 3900,
Ribeirao Preto, São Paulo 14049-900, Brazil.
Tel: +55 16 3315-3181
e-mail: rtostes@usp.br / nathanneferre@hotmail.com

SUPPLEMENTARY METHODS

Oral Glucose Tolerance Test

Control and type 2 diabetes mice were treated with the NLRP3 selective inhibitor MCC950 (10 mg/kg/ day, intraperitoneal injections) or respective vehicle for 2 weeks and at the end of the treatment, the animals were fasted for 8 h and fasting glycaemia was determined, characterizing the time 0. After fasting glucose determination, a glucose overload at the dose of 2 g/kg was administered, per gavage, to the animals. New blood samples were taken to determine the blood glucose at 15, 30, 60, 90 and 120 minutes (min) after glucose administration. Blood was collected after cutting at the tip of the tail and placed in individual tapes and the reading was performed on a glucometer Accu-Chek Active® (Roche Diagnostics, Mannheim, Germany).

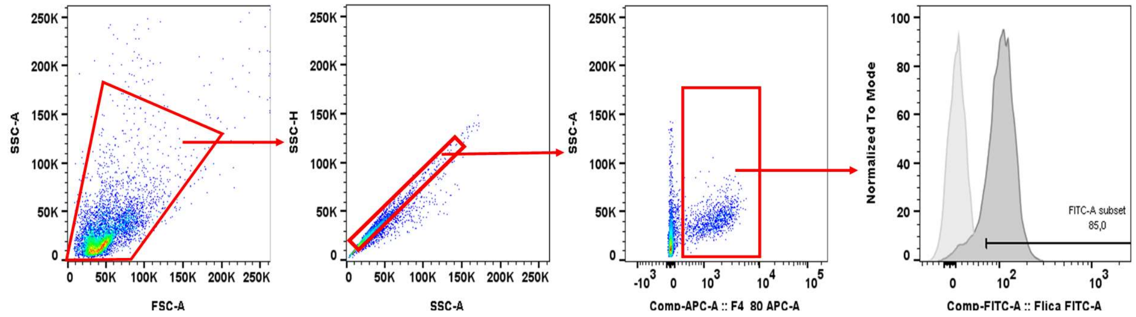

SUPPLEMENTARY FIGURE 1. Flow cytometry gating strategy for caspase-1 activity in macrophages of the peritoneal lavage. Activity of caspase-1 in macrophages was determined by flow cytometry in the peritoneal lavage of control and db/db mice treated with vehicle or spironolactone for 6 weeks as indicated in the text. Total cells were stained with allophycocyanin (APC) and FAM-FLICA® Caspase-1 (FAM-YVAD-FMK). Fluorophores were respectively excited and analyzed with the appropriate laser and band pass filter (BP): (FITC 492 nm with 520 nm and APC 633-647 nm with 660 nm). A representative Flow Cytometry profile of macrophages in db/db mice vehicle after 6 weeks of treatment demonstrating the gating strategy. The macrophages were gated in the side scatter area (SSC-A)/forward scatter area (FSC-A) plot. Singlet cells were gated using SSC-A over SSC-wide (SSC-W). Macrophages (F4/80$^+$) were gated in the SSC-A and positive caspase-1 were in histogram from the F4/80$^+$.

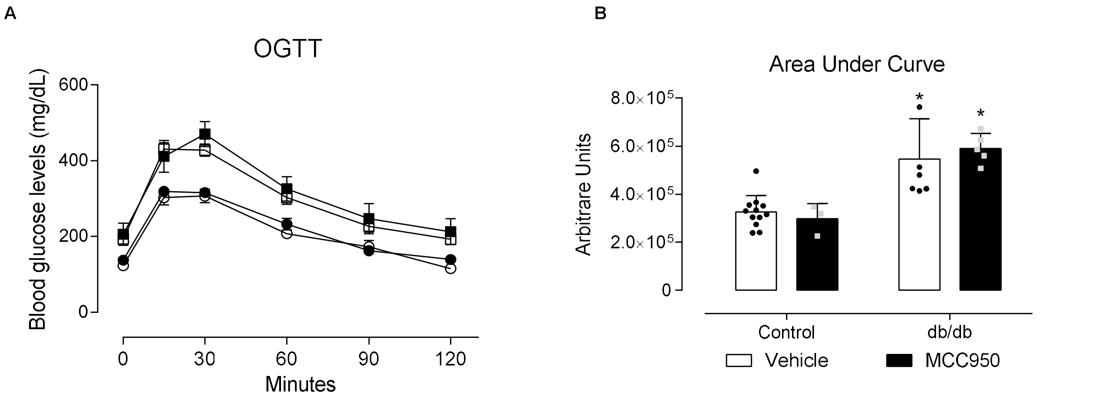

SUPPLEMENTARY FIGURE 2. Treatment with the NLRP3 inhibitor does not alter glucose tolerance in db/db mice. Oral Glucose Tolerance Test (OGTT) (A) and Area Under Curve of the Oral Glucose Tolerance Test (B) in control and db/db mice treated with vehicle or MCC950 (10 mg/Kg/day) for 2 weeks (n=3-7 mice per group). Data represent the mean ± S.E.M. Two-way ANOVA with Bonferroni post-test, $p < 0.05$ * *vs*. control.

SUPPLEMENTARY TABLE S1. pD_2 values of PE-induced maximal contraction and ACh-induced relaxation in mesenteric arteries of diabetic and control mice treated or incubated with vehicle spironolactone or MCC950.

	**Control**		**db/db**	
	***p*D2 PE**	***p*D2 ACh**	***p*D2 PE**	***p*D2 ACh**
**Vehicle**	6.7 ± 0.06	7.2 ± 0.15	6.6 ± 0.08$^{\&}$	6.2 ± 0.29*
	n=8	n=6	n=9	n=7
**Spironolactone (6 weeks)**	6.2 ± 0.12*	7.4 ± 0.16	6.3 ± 0.07	7.1 ± 0.22†
	n=4	n=4	n=5	n=4
**Vehicle**	6.6 ± 0.08	7,3 ± 0.07	6.7 ± 0.15	6.7 ± 0.19*
	n=6	n=10	n=6	n=10
**MCC950 (1 hour)**	6.1 ± 0.06*†	7,1 ± 0.07	6.7 ± 0.08	7.1 ± 0.18
	n=6	n=6	n=4	n=4
**Vehicle**	6.6 ± 0.12	7.3 ± 0.14	6.6 ± 0.06	6.6 ± 0.26
	n=5	n=4	n=4	n=5
**MCC950 (2 weeks)**	6.7 ± 0.08	7.5 ± 0.07$^{\#}$	6.5 ± 0.09	7.2 ± 0.14
	n=4	n=4	n=5	n=5

Data represent the mean ± S.E.M (n= 4-10 mice per group). Two-way ANOVA with Bonferroni post-test, $p < 0.05$ * *vs*. control vehicle; & *vs*. Control Spironolactone; † *vs*. db/db vehicle and # *vs*. db/db vehicle. MCC950: NLRP3 inhibitor, PE: phenylephrine, ACh: acetylcholine.

SUPPLEMENTARY TABLE S2. Maximal responses to PE and ACh in mesenteric arteries from control and db/db mice treated with spironolactone or MCC950 and in arteries from control mice incubated with vehicle or NLRP3 inhibitor or aldosterone.

	**Control**		**db/db**	
	**Rmax PE**	**Rmax ACh**	**Rmax PE**	**Rmax ACh**
**Vehicle**	134.6 ± 3.2	76.4 ± 3.8	141.7 ± 4.7	53.4 ± 6.1*
	n=8	n=6	n=9	n=7
**Spironolactone (6 weeks)**	138.6 ± 7.1	75.3 ± 4.5	129.8± 3.9	63.8± 4.7
	n=4	n=4	n=4	n=4
**Vehicle**	153.8 ± 5.3	91.3 ± 2.3	154.3 ± 9.7	44.1 ± 3.2*
	n=6	n=10	n=6	n=10
**MCC950 (1 hour)**	142.5 ± 4.3	95.9 ± 2.5	150.3 ± 5.5	71.9 ± 4.5*†
	n=6	n=6	n=4	n=4
**Vehicle**	137.6 ± 6.6	89.4 ± 3.9	133.0 ± 3.5	52.1 ± 4.9*

	n=5	n=4	n=5	n=5
**MCC950**	146.4 ± 4.8	98.4 ± 2.3	135.1 ± 5.2	83.5 ± 4.0†
**(2 weeks)**	n=4	n=4	n=5	n=5

Data represent the mean ± S.E.M (n= 4-10 mice per group). Two-way ANOVA with Bonferroni post-test, $p < 0.05$ * *vs*. control vehicle; and †*vs*. db/db vehicle. MCC950: NLRP3 inhibitor, PE: phenylephrine, ACh: acetylcholine.
